# Editorial Items

**Published:** 1884-08

**Authors:** 


### EDITORIAL ITEMS.

Dr. H. V. M. Miller left the city a few days ago for Waynesville, North Carolina. He will be absent for several months. He was accompanied by Mrs. Miller.

Dr. J. S. Todd is building a $15,000 residence on Marietta street, near the Capitol. It will be completed by October 1st.

Dr. George H. Noble and wife have returned to the city, after a pleasant visit to relatives in Anniston, Alabama.

Dr. R. O. Cotter, who has been in bad health for several weeks, has gone to the country to spend a short time.

The Atlanta Medical and Surgical Union is the name of a new medical society organized here not long since. Dr. W. S. Elkin is the efficient president.

We understand that a Surgical Infirmary will be organized here in a few weeks with one of the most prominent surgeons in the State at the head of it. Such an institution is very much needed in this city.

Dr. J. McF. Gaston now occupies the office vacated by Dr. A. W. Calhoun, No. 38½ South Broad street. Dr. Calhoun has removed to his elegant office on Marietta street, near the Capitol.

It gives us pleasure to note the fact that Dr. Robert Battey, of

Rome, is having the very best results with his Operation. He has recently made Battey's operation on thirty-four cases without a death.

We have received a beautiful picture of the Southern Exposition, which opens at Louisville, Ky., August 16th, and continues until October 25th. The view is of the main building, which is one of the largest exposition buildings ever erected. It covers thirteen acres of ground, and will be lighted throughout by five thousand electric lights.

NEW JOURNAL.—We are in receipt of the first number of the *American Journal of Ophthalmology.* This is published by J. H. Chambers & Co., of St. Louis, and is the only monthly published in the United States on this subject. It is edited by Adolph Alt, M.D., and presents a fine appearance. Pages, 32 ; $2.50 per annum.

Our senior Senator, Gov. Jos. E. Brown, has placed us under obligations for valuable public documents and other favors.

Gov. Brown has always had much opposition in Georgia, it is but natural that a man of his positive character should, but his most bitter opponents cannot gainsay the fact that his record as a senator, covering now about five years, has proved him very able and conservative. He has especially looked carefully after the material interests of the State, and aided largely in obtaining many large appropriations for our rivers and harbors. And when he has spoken in debate no one has overcome him. As Artemus Ward would say, "On the contrary, quite the reverse."

It now seems almost certain that he will be re-elected by the incoming Legislature without opposition, and perhaps this will increase his power to be useful and do his State valuable service.

The Alumni Association of Jefferson Medical College has inaugurated a movement to secure in some medical school the endowment of a memorial professorship to be designated the S. D. Gross professorship of pathological anatomy. The profession everywhere is invited to contribute to this fund. Contributions may be sent to Dr. R. J. Dunglison, lock box 1274, Philadelphia, or to Drs. W. F. Westmoreland, A. W. Calhoun, Atlanta; H. F. Campbell, Augusta; Robt. Battey, Rome, or A. J. Semmes, Savannah.

Dr. Thos. L. Raines, a young man of more than ordinary promise, died at his home in this city on the morning of July 20th. A more extended notice of his death will appear in our next issue.

Dr. Wesley C. Norwood, long one of the most eminent physicians of South Carolina, and a medical writer of note, died on the 15th of July, at his home in Cokesbury, S. C., at the ripe age of 78. He was the discover of veratrum viride.

